# Spinal versus general anesthesia for hip arthroscopy—a pandemic (COVID) and epidemic (opioid) driven study

**DOI:** 10.1093/jhps/hnae009

**Published:** 2024-03-27

**Authors:** J. W. Thomas Byrd, Kay S Jones, Nicole Dwyer, Amy M McManus, Ellen B Byrd, Wallace L Freeman

**Affiliations:** Nashville Sports Medicine Foundation, 2004 Hayes Street, Suite 700, Nashville, TN 37203, USA; Nashville Sports Medicine Foundation, 2004 Hayes Street, Suite 700, Nashville, TN 37203, USA; USPI, 2004 Hayes Street, Suite 450, Nashville, TN 37203, USA; USPI, 2004 Hayes Street, Suite 450, Nashville, TN 37203, USA; Nashville Sports Medicine Foundation, 2004 Hayes Street, Suite 700, Nashville, TN 37203, USA; Anesthesia Medical Group, 110 29th Ave. North, Suite 202, Nashville, TN 37203, USA

## Abstract

The purpose of this study is to compare general anesthesia (GA) to spinal anesthesia (SA) for hip arthroscopy, based on measurable perioperative parameters. The pandemic signaled a change from GA to SA, and thus a retrospective review was performed of the first 120 consecutive SA cases compared to the last 120 GA cases prior to the pandemic. Demographic data included age, sex, BMI, preop narcotic usage and procedure performed. The groups were compared for post-anesthesia care unit length of stay, entry and discharge visual analog scale (VAS) scores, morphine mg equivalent usage, need for regional blocks and untoward events. Additionally, the length of time from entry to the operating room until completion of induction anesthesia was compared. Demographically, the groups were virtually identical. SA used significantly less morphine mg equivalent (6.0 versus 8.1; *P* = 0.005), had more needing no narcotics (17 versus 7; *P* = 0.031), fewer requiring blocks (1 versus 14; *P* = 0.001) and lower entry VAS scores (5.2 versus 6.2; *P* = 0.003). Five early SA patients required catheterization for urinary retention, and this was avoided later in the study by having patient void on call to operating room and avoiding anticholinergic agents. Completion of induction anesthesia was 0.8 min longer for SA. Hip arthroscopy can be effectively performed with either GA or SA. SA results in statistically significant better post-anesthesia care unit pain control, reflected by lower entry VAS, less need for narcotics and fewer requiring regional blocks compared to GA. Urinary retention, a potential problem of SA, is minimized with routine precautions.

## INTRODUCTION

As a backdrop, general anesthesia (GA), including a combination of intravenous and inhalational agents, is the most common anesthesia mode used for hip arthroplasty. This is even in light of substantial literature, reflecting fewer complications and better outcomes with neuraxial anesthesia, most commonly spinal anesthesia (SA) [1–5].

Little attention has been given to the mode of anesthesia for hip arthroscopy with most focusing on the role of regional blocks for postoperative pain control [6–10]. Only the recently published work by Turner *et al*. looked at neuraxial compared to GA [11]. Hip arthroscopy patients generally represent a younger population than arthroplasty patients and likely have fewer comorbidities to be considered.

For 30 years, the senior author had always used GA for hip arthoscopy [12]. This was also routinely performed as an outpatient procedure.

With resumption of non-urgent surgery following the early phases of the COVID (coronovirus disease) pandemic, SA became the standard for hip arthroscopy at Nashville Hip Institute. The reasoning was to reduce potential exposure to the virus associated with patient intubation and to reduce consumption of scarce personal protective equipment supplies [13–15]. Given that a unilateral shift had occurred from GA to SA, it was hypothesized that SA could prove to have comparable efficacy. The purpose of this study is to compare GA to SA based on measurable perioperative parameters.

## MATERIALS AND METHODS

Since 1990, hip arthroscopy has routinely been performed as an outpatient procedure under GA with endotracheal intubation, incorporating muscle relaxation as an adjunct for optimizing distractibility of the joint [12]. All procedures have been performed on the same fracture table with the supine technique [16]. Postoperative rehabilitation has begun the day following surgery, typically with crutches 50% weightbearing for the first 4 weeks [17]. These procedures ceased in March 2020 as part of the suspension of all non-urgent surgery in response to the COVID pandemic. Upon resumption of these procedures in May 2020, the decision was made to use SA as the standard anesthesia. Contraindications to SA were previous lumbar spine surgery, BMI >35 or patient refusal.

The study consists of the first consecutive patients undergoing SA following resumption of surgery compared to an equal number of the last patients immediately prior to the pandemic undergoing GA (Table I). This study received exemption status from the institutional review board.

**Table I.** *Protocol common to both GA and SA*

*Preop*	*Intraop*	*Postop*
• Decadron 4 mg orally	• Versed 2 mg intravenous (IV)	PONV protocol Pro Re Nata (PRN):
	• Fentanyl 50–100 μg IV	• Decadron
• Aspirin 81 mg orally		• Zofran
	Diprivan bolus and infusion	• Haldol 1 mg IV
		• Fluids
	BP control:	
	• Labetalol 5–20 mg IV	Pain management PRN:
	• Enalapril 0.25–1.25 mg IV	• Fentanyl 25–100 μg IV
	• Cardene infusion	• Dilauded 0.25–1.0 mg IV
	PONV prevention:	Regional block PRN^a^:
	• Zofran 4 mg IV	• LFCNB
		• FICB
	Pain control:	• FNB
	• Toradol 30 mg IV	
		Oral meds PRN:
	Hip joint injection by surgeon:	• Percocet 5/325 mg
	• Marcaine 0.25%	• Lortab 7.5/325 mg
		• Oxycodone 5 mg
		• Gabapentin 100–300 mg
GA protocol		
• Antivert 12.5 mg orally	• Diprivan induction dose	
• Phenergan 12.5 mg orally	200 mg IV	
• Scopolamine patch 0.4 mg	• Intubation with endotracheal tube	
• Pepcid 20 mg orally	Muscle relaxant	
	• Succinylcholine 100–140 mg IV	
	• Rocuronium 30–50 mg	
	Inhalational anesthetic:	
	• Forane	
	• Sevoflurane	
	Muscle relaxant reversal:	
	• Neostigmine 1–5 mg IV	
	• Robinol 0.1–0.5 mg IV	
	• Suggamadex 100–200 mg IV	
SA protocol		
• Flomax 0.4 mg orally	SA:	Urinary retention protocol:
	• Pencan 25 g 3.5″ needle	• Repeat Flomax
	or Pencan 24 g 4.0″ needle	• Neostigmine 1 mg intramuscular (IM)
	• Mepivacaine MPF 2%, 2.6–3.4 ml	• Ephedrine 10 mg IM
	or bupivacaine 0.75%, 1.6–2.0 ml	
	Diprivan infusion	
	Precedex infusion	

a Regional block utilized when pain uncontrolled with IV medication. Type of block selected based on character and location of pain.

Abbreviations: PONV, postop nausea and vomiting; BP, blood pressure; LFCNB, lateral femoral cutaneous nerve block; FICB: fascia iliaca compartment block.

Post-anesthesia care unit (PACU) parameters studied included length of stay (LOS), entry and discharge visual analog scale (VAS) score, morphine milligram equivalent (MME) usage, need for regional blocks and untoward events. Additionally, the length of time from entry to the operating room (OR) until completion of anesthesia was compared. A power analysis, based on MME, calculated that 112 patients in each group were necessary to determine statistical significance, and this was rounded up to 120 patients in each group. Of note, during the collection period of 120 SA patients, 41 patients underwent GA in whom SA was contraindicated.

Independent samples *t*-tests, chi-square tests and Mann–Whitney U tests were used where appropriate. Significant results were determined by *P* < 0.05.

## RESULTS

The demographics of the two study populations were virtually identical (Table II). Significant differences included: SA patients using less MME (6.0 versus 8.1; *P *= 0.005); more needing no narcotics 14.2% (*n* = 17) versus 5.8% (*n* = 7) (*P* = 0.031); fewer requiring regional blocks 0.89% (*n* = 1) versus 11.7% (*n* = 14) (*P* = 0.001); and lower entry VAS scores (5.2 versus 6.2; *P* = 0.003) compared to GA (Table III). There was less nausea in the SA group [3.3% (*n* = 4) versus 9.2% (*n* = 11)], but the difference was not statistically significant (*P* = 0.124). Five (4.2%) SA patients required catheterization for urinary retention (Table IV). Four occurred among the first 11 patients, and this was subsequently reduced by having the patient void on call to the OR and avoiding anti-cholinergic agents. One (0.8%) SA was ineffectual, requiring conversion to GA, and one (0.8%) patient developed a spinal headache. Completion of induction anesthesia was 0.8 min longer for SA, which was statistically significant (*P* = 0.005): SA 8.4 min (range 5–16) versus GA 7.6 min (range 5–14).

**Table II.** *Comparative demographics*

*Patient demographics*	*SA*	*GA*	P*-value*
Age	31 (range 15–60)	31 (range 13–72)	0.992
Female/male (ratio)	84/36 (2.3)	80/40 (2.0)	0.579
BMI	25 (range 18–38)	26 (range 18–42)	1.000
Opioid naïve	111 (92.5%)	108 (90%)	0.493
FAI surgery	105	105	1.000
Revision surgery	12	10	0.655

**Table III.** *PACU parameters*

	*SA*	*GA*	P-*value*
LOS	189 min (range 72–574)	195 min (range 76–463)	0.501
Admission VAS	5.2 (range 0–10)	6.2 (range 0–10)	0.003
Discharge VAS	3.0 (range 0–6)	2.9 (range 0–7)	0.484
MME usage	6.0 (range 0–17.7)	8.1 (range 0–37.9)	0.005
No narcotics	17	7	0.031
Regional blocks	1 LFCNB	14 (11 FICB; 3 FICB + LFCNB)	0.001

**Table IV.** *Untoward events*

	*SA*	*GA*	P-*value*
Nausea requiring treatment	4	11	0.124
Urinary retention	5	0	0.029

## DISCUSSION

While the principal observation of this report is that SA results in better PACU pain control than GA, of equal significance is the historical circumstances that spawned this study. The catalyst for this conversion from GA to SA was the COVID pandemic. The concern of the potential virus exposure to healthcare workers associated with endotracheal intubation has been substantiated in the literature [13–15]. Conversion to SA seemed appropriate in terms of optimizing patient care based on extensive literature supporting the favorable profile of SA compared to GA [1–5]. Indirectly, patient care was enhanced by preserving care access via lessening exposure of healthcare workers and lessening consumption of personal protective equipment associated with endotracheal intubation [15]. Serendipitously, this provided an opportunity to compare SA versus GA for hip arthroscopy.

SA represents a favorable immediate postop pain control profile compared to GA as reflected by statistically significantly lower PACU entry VAS scores, less MME usage, more patients requiring no narcotics and fewer patients requiring regional blocks.

The LOS in the PACU was less with SA, but the difference was not statistically significant. Additionally, the lower VAS score associated with SA was no longer statistically significant by the time of discharge. However, it is also worthy to note that SA does not prolong LOS in the PACU and does not delay discharge.

The favorable influence of SA on pain control cannot be overemphasized. With the unveiling of the opioid crisis, it is known that exposure to opioids in the perioperative period increases the risk of chronic opioid dependance [18–21]. In fact, patients undergoing hip arthroscopy may be particularly vulnerable to opioid exposure even prior to surgery. This is due to the sometimes elusive nature of hip disorders amenable to arthroscopy. In an earlier study from this center, it was noted that 60% of athletes undergoing hip arthroscopy had been treated for an average of 7 months before it was recognized that the joint was the source of the problem [22]. In a similar vein, Clohisy *et al*. also noted that young adults with non-arthritic hip disorders saw an average of 4.2 healthcare providers before a diagnosis was established [23]. It would not be uncommon that with little else to offer for these poorly explained symptoms, prescription analgesics may be a seemingly simple solution.

Also of note, it is preferred to avoid using regional blocks. An observation of this center is that regional blocks can significantly interfere with the early postoperative rehabilitation process, which generally begins the day following surgery. While some studies have reflected benefits of immediate postop pain control, others have questioned the positive effect of regional blocks, and complications, especially falls, may discourage routine use [7–10].

SA added 0.8 min to the beginning of the case, compared to GA, and while the difference was statistically significant, the clinical relevance is just slight. A meaningful observation which was not part of this study was that the turnaround time between cases was lessened since it was not necessary to wait for the patient to be awakened and be extubated before transfer to the PACU. The high number of urinary retention cases early in the SA experience reflects the learning curve that was subsequently corrected.

The observations on pain from this study are quite comparable to those of Turner *et al*., which is the only other publication comparing neuraxial (SA or epidural) to GA for hip arthroscopy. They reported on 129 patients (77 neuraxial; 52 GA), allocated based on the patient’s preference and found lower immediate postop pain scores and opioid needs with neuraxial anesthesia. However, their total LOS was 42 min longer with neuraxial anesthesia, and while this was attributed to the SA or epidural being performed in the preoperative holding area, it could reflect a meaningful disadvantage [11]. This is contrary to the observations of this present study where the SAs are performed in the OR, adding only 0.8 min, and the PACU LOS was slightly shorter. Thus, the total LOS was not any longer.

A few further practical observations that would not be reflected in this data warrant comment. In this small private practice setting, intimate teamwork is an essential element in the continuity of care provided for patients. With GA, the nurse anesthetist is the most integrative member of the anesthesia team as they perform the intubation and prepare the patient for surgery. SAs are performed by the anesthesiologist, and thus, with SA, the anesthesiologist becomes a much more integrated part of the team. Knowing the abilities of the individual performing the SA becomes just as important as who is managing the anesthesia during the procedure.

SA also requires more duties on the part of the office staff. Preoperative screening laboratories for SA are not needed for GA. Scrutiny of the patient’s medical history also becomes more relevant in the decision for SA in addition to orienting the patient and family to the expectations of SA. At this center, the charges for GA and SA were the same.

As a non-randomized study, the principal limitation of this methodology is that the two groups are sequential and not simultaneous. However, since there was a moment in time when the switch was made from GA to SA, this minimizes other uncertain variables that might be associated with progressive experience or other changes over time. Of particular importance, this study represents simply a snapshot of what occurs in the OR and immediately afterwards in the PACU. No extrapolation is attempted on how this might positively or negatively influence each individual’s subsequent pain control needs. Also of note, this is the experience of a single outpatient surgery center and may not be generalizable to other centers.

## CONCLUSION

Hip arthroscopy can be effectively performed with either GA or SA. SA results in statistically significant better PACU pain control, reflected by a lower entry VAS, less need for narcotics and fewer requiring regional blocks compared to GA. Urinary retention, a potential problem of SA, is minimized with routine precautions.

## Data Availability

The data underlying this article will be shared on reasonable request to the corresponding author.
