# Correction to: Spinal versus general anesthesia for hip arthroscopy—a pandemic (COVID) and epidemic (opioid) driven study

**DOI:** 10.1093/jhps/hnae022

**Published:** 2024-06-19

This is a correction to: JW Thomas Byrd *et al*., Spinal versus general anesthesia for hip arthroscopy—a pandemic (COVID) and epidemic (opioid) driven study, *Journal of Hip Preservation Surgery*, 2024; hnae009, https://doi.org/10.1093/jhps/hnae009

The first author’s name has been corrected.

